# The clinical application of subcutaneous thoracic ratio and capillary leakage index on the occurrence of capillary leak syndrome in neonates with sepsis

**DOI:** 10.3389/fped.2025.1603378

**Published:** 2025-07-07

**Authors:** Luying Cao, Yuhong Song, Li Zhang, Xiaolu Liu, Yiying Yin, Zhenrong Yu, Yu Zhang, Kun Feng, Weihong Yue, Ya Hu, Ziyu Hua, Hong Wei

**Affiliations:** Department of Neonatology, National Clinical Research Center for Child Health and Disorders, Ministry of Education Key Laboratory of Child Development and Disorders, Chongqing Key Laboratory of Pediatric Metabolism and Inflammatory Diseases, Children’s Hospital of Chongqing Medical University, Chongqing, China

**Keywords:** capillary leak syndrome, capillary leakage index, neonate, sepsis, subcutaneous thoracic ratio

## Abstract

**Objective:**

To evaluate the predictive and prognostic value of the subcutaneous-thoracic ratio (S/T) and capillary leakage index (CLI) for capillary leak syndrome (CLS) in neonatal sepsis.

**Materials and methods:**

A cohort of 196 neonates with sepsis, admitted to a tertiary children's hospital in southwestern China between January 2019 and March 2021, was included in the study. The neonates were divided into two groups: the CLS group (*n* = 55) and the non-CLS group (*n* = 55). Multivariate logistic regression and receiver operating characteristic (ROC) curve analysis were performed to identify key predictors of CLS.

**Results:**

Both S/T and CLI were found to be independent risk factors for CLS in neonatal sepsis (*P* < 0.05). The median S/T values for the CLS group and non-CLS group were 9.0% and 7.1%, respectively, while the median CLI values were 8.5 and 3.2. The optimal thresholds for predicting CLS were identified as 8.1% for S/T (sensitivity: 67.3%, specificity: 70.9%) and 3.3 for CLI (sensitivity: 78.2%, specificity: 56.4%). Notably, the combination of S/T and CLI yielded improved predictive performance, with a sensitivity of 81.8% and specificity of 60.0%. However, neither S/T nor CLI were significantly associated with prognosis, as no difference was observed between survivors and non-survivors (*P* > 0.05).

**Conclusion:**

The combined application of S/T and CLI provides an effective tool for predicting the occurrence of CLS in neonatal sepsis. However, these indicators do not demonstrate prognostic value for survival outcomes.

## 1 Introduction

Capillary leak syndrome (CLS), first described by Clarkson in 1960, is a rare but severe condition characterized by widespread capillary hyperpermeability, leading to fluid and protein leakage from the intravascular space into the interstitial tissues (1). This results in hypovolemia, hemoconcentration, hypoalbuminemia, systemic edema, and weight gain, among other systemic manifestations (2). CLS is typically precipitated by systemic inflammation, which causes damage to the endothelial structure and integrity of blood vessels. Neonatal CLS is particularly concerning due to its rapid progression and potential for multi-organ dysfunction, which significantly increases mortality rates if not promptly identified and treated (3–5). Despite the grave prognosis associated with neonatal CLS, its exact pathophysiology remains poorly understood, and the diagnosis is frequently delayed.

In neonates, sepsis is a major risk factor for the development of CLS, with an estimated incidence rate of 4.5‰–9.7‰ (6). The clinical presentation of neonatal CLS is often nonspecific, with signs such as edema, decreased urine output, and respiratory distress (7). These clinical features overlap with other neonatal conditions, making early diagnosis challenging. Moreover, the lack of widely accessible diagnostic tools further complicates the management of CLS in neonates. Traditional diagnostic methods, such as extracellular inulin distribution measurement and bioelectrical impedance analysis, are considered gold standards for assessing capillary leakage. However, these techniques are impractical for routine clinical use due to their high cost, complexity, and need for specialized equipment (8). As such, there is an urgent need to identify reliable, cost-effective, easily measurable biomarkers or indices to predict and assess the severity of CLS in neonates.

In this context, recent studies have highlighted the potential of the subcutaneous-thoracic ratio (S/T) as a predictive indicator for CLS in neonates. The S/T ratio, first introduced by Sonntag in 2003, quantifies the soft tissue thickness relative to the thoracic diameter at the level of the eighth rib on neonatal chest radiographs (9). Despite its potential, clinical research on the S/T ratio in neonatal CLS remains sparse, with few studies validating its use in routine clinical practice. Another promising indicator for predicting and assessing CLS is the capillary leakage index (CLI), defined as the ratio of C-reactive protein (CRP) to albumin. The CLI reflects both the inflammatory state and the permeability of the microvascular endothelium, with higher CLI values suggesting more severe capillary leakage. However, the clinical utility of CLI as a diagnostic and prognostic marker in neonates remains underexplored, especially in the context of neonatal sepsis.

Despite the potential of both S/T and CLI as clinical indicator, no study has yet assessed their combined predictive value in the context of neonatal CLS. Therefore, the primary aim of this retrospective cohort study is to evaluate the value of the S/T ratio and CLI in predicting the occurrence and severity of CLS in neonates with sepsis. By combining these two indices, we hope to improve the accuracy of early diagnosis and risk stratification for CLS, ultimately leading to better management strategies and improved outcomes for neonates affected by this life-threatening condition.

## 2 Materials and methods

### 2.1 Study subjects

A total of 196 neonates diagnosed with sepsis and admitted to a tertiary children's hospital in southwestern China between January 2019 and March 2021 were included in this study. Idiopathic CLS, with different etiologies, was not included in this study (10). Baseline data, laboratory parameters, imaging results, treatment modalities, and clinical outcomes were extracted from the hospital's medical records. The cohort consisted of 59 neonates with CLS and 137 neonates without CLS. After propensity score matching (PSM), 110 neonates were selected (55 matched pairs). PSM was conducted using a 1:1 ratio with a caliper value of 0.02 to balance baseline characteristics (gender, gestational age, birth weight, age, delivery method) between the two groups. Infants in the CLS group were subdivided into survival and death groups based on their clinical outcomes at 28 days (alive or deceased). The study was approved by the Institutional Review Board of the hospital (Approval No. 2021-273), with a waiver of informed consent granted due to the retrospective nature of the study.

### 2.2 Inclusion and exclusion criteria

Diagnostic criteria for neonatal sepsis (11, 12): (1) Clinical manifestations with nonspecific signs, such as temperature instability, respiratory issues, lethargy, or feeding problems, progressing to severe complications, such as multiple organ failure (MOF) and septic shock; (2) At least one of the following: Two or more positive blood non-specific inflammatory markers; Cerebrospinal fluid examination showing purulent meningitis; Positive blood culture or detection of pathogenic bacteria DNA.

Inclusion criteria for neonates in the CLS group: (1) Admitted at ≤ 28 days of age; (2) Chest radiography was performed before generalized edema. (3) Clinical diagnosis of CLS based on the following: (a) Secondary to neonatal sepsis; (b) Symptoms: generalized edema, hypovolemic hypotension, oliguria/anuria; (c) Laboratory findings: hypoalbuminemia and hemoconcentration; (d) Positive liquid replenishment test: worsening of edema following crystalloid infusion; (e) Exclusion of cardiogenic, hepatogenic, nephrogenic, and inherited metabolic causes of edema (5, 10, 13).

Exclusion criteria: (1) Incomplete clinical or laboratory information; (2) Thoracic and limb deformities or inherited metabolic diseases; (3) Generalized edema present before hospital admission; (4) Death or discharge within 48 h of admission; (5) These cases met the inclusion criteria of the CLS group, but there were no matched cases in the control group.

### 2.3 Observation indicators

All patients underwent laboratory tests and chest radiography within 72 h of admission, with follow-up assessments guided by clinical progression.

CLS group: The most recent indicator prior to the onset of generalized edema was included.

Non-CLS group: Indicators within 72 h of admission were included.

### 2.4 Statistical analysis

The two indicators, CLI and S/T, were calculated as follows:

(1) CLI = C-reactive protein (mg/dl) ÷ albumin (g/L) × 100;
(2) S/T = 100%—distance between the outer margins of the eighth rib ÷ total thoracic diameter at the same position (Supplementary Figures S1, S2 in Supplementary Material).

Statistical analyses were conducted using SPSS version 25.0 (IBM). Continuous variables were expressed as means ± standard deviation (SD) for normally distributed data, or as medians with interquartile ranges (IQR) [M (P25, P75)] for skewed data. Categorical data were compared using the Chi-square test or Fisher's exact test. Differences between groups for continuous variables were assessed using the independent *t*-test for normally distributed data and the *Mann–Whitney U-*test for non-normally distributed data. Predictors of CLS were entered into multivariate logistic regression model to identify independent associations and calculate odds ratios (OR) with 95% confidence intervals (CIs). Receiver operating characteristic curves (ROC) and the area under the curve (AUC) were constructed to assess the predictive accuracy of CLI and S/T for the occurrence of CLS. *P* < 0.05 was considered statistically significant.

## 3 Results

### 3.1 Baseline characteristics

Among the 196 neonates with sepsis, 124 (63.3%) were male and 72 (36.7%) were female. The mean gestational age was 34.32 ± 3.98 weeks, and the mean birth weight was 2,188 ± 895 grams. In this study, we classified neonatal sepsis into early-onset sepsis (EOS) and late-onset sepsis (LOS) based on the age of onset. EOS was defined as sepsis occurring within the first 72 h of life, whereas LOS occurred after 72 h (12, 14). The average sepsis onset age for the entire cohort was 1.3 ± 0.8 days, which suggests that all of the cases in our study were EOS. After PSM, 55 pairs of infants were matched for clinical data analysis and divided into the CLS group (*n* = 55) and the non-CLS group (*n* = 55). No significant differences in baseline characteristics were observed between the two groups (*P* > 0.05) (Table 1).

**Table 1:** Baseline characteristics after propensity score analysis.

Variables^†^	CLS (*n* = 55)	Non-CLS (*n* = 55)	*P*-value
Sepsis onset age (days)	1.0 (1.0, 1.0)	1.0 (1.0, 1.0)	0.195
Gestational age (weeks)	33.4 (30.2, 37.3)	33.7 (31.0, 38.6)	0.495
Birth weight (g)	2,060 (1,400, 2,900)	1,940 (1,370, 3,200)	0.496
Genders, *n* (%)			
Male	39 (70.9)	43 (78.2)	0.381
Female	16 (29.1)	12 (21.8)	
Delivery method, *n* (%)			
Vaginal delivery	11 (20.0)	12 (21.8)	0.815
Cesarean section	44 (80.0)	43 (78.2)	
Singleton, *n* (%)	46 (83.6)	46 (83.6)	1.000
Gestational diabetes mellitus, *n* (%)	20 (36.4)	16 (29.1)	0.416
Infection during pregnancy, *n* (%)	14 (25.4)	20 (36.4)	0.216
Intrauterine distress, *n* (%)	18 (32.7)	10 (18.2)	0.080

^†^ Variables as number (*n*%) or median [inter-quartile range].

### 3.2 Analysis of clinical conditions

In the CLS group, 85.4% of cases presented with generalized edema, and 14.5% had localized edema. The median onset time of generalized edema was 3 (IQR: 2, 4) days after admission. There were 15 (27.3%) cases of abdominal effusion, 4 (7.3%) cases of pleural effusion, and 1 (1.8%) case of pericardial effusion. Edema lasted a median of 5 (IQR: 2, 7) days in survivors but persisted in those who died. The CLS group demonstrated a significantly higher incidence of severe complications compared to the non-CLS group. Shock was observed in 43.6% of CLS cases compared to only 10.9% in the non-CLS group (*P* < 0.0001), indicating pronounced circulatory instability in CLS patients. Pulmonary hemorrhage was notably more frequent in the CLS group (52.7% vs. 12.7%, *P* < 0.0001), highlighting the vulnerability of these patients to severe respiratory complications. Electrolyte imbalances such as hypokalemia (16.4% vs. 0.0%, *P* = 0.003) and hematologic issues like thrombocytopenia (16.4% vs. 3.6%, *P* = 0.026) were also significantly more common in the CLS group. Furthermore, gastrointestinal infections were observed in 20.0% of CLS patients compared to 5.4% in the non-CLS group (*P* = 0.045), reflecting a greater susceptibility to systemic infections (Supplementary Table S1). Although renal insufficiency, liver dysfunction, myocardial damage, and intracranial hemorrhage were more frequently reported in the CLS group, these differences were not statistically significant.

Treatment requirements and clinical outcomes between the two groups also differed substantially (Supplementary Table S2). Mechanical ventilation was almost universal in the CLS group (98.2% vs. 76.4%, *P* = 0.002), with a significantly longer duration (median 8.5 vs. 5.0 days, *P* = 0.006), reflecting the severity of respiratory compromise. The administration of blood products and albumin was much higher in the CLS group, with 90.9% receiving blood products compared to 54.5% in the non-CLS group (*P* < 0.0001), and the median amount of albumin administered was significantly greater (11.6 g vs. 2.4 g, *P* < 0.0001). Vasoactive drugs were also used more frequently in the CLS group (85.4% vs. 40.0%, *P* < 0.0001), further emphasizing the critical condition of these patients. Despite these differences in complications and treatments, there was no statistically significant difference in hospitalization duration (24.0 vs. 27.0 days, *P* = 0.848) or mortality rate (18.2% vs. 7.3%, *P* = 0.153) between the two groups. This finding suggests that while CLS patients experienced more severe clinical manifestations and required more intensive treatments, the outcomes in terms of mortality and length of hospital stay were comparable, potentially reflecting the effectiveness of interventions in mitigating the adverse impacts of CLS.

### 3.3 Predictors for the occurrence of CLS

Comparison between the two groups revealed that the S/T, CLI, CRP, coagulation disorders, blood glucose, and blood lactate levels were significantly higher in the CLS group than in the non-CLS group (*P* < 0.05), while the albumin level was significantly lower in the CLS group (*P* < 0.05). There were no statistically significant differences between the two groups in procalcitonin, white blood cell count, serum calcium, or red blood cell distribution width (*P* > 0.05, Table 2).

**Table 2:** Comparison of laboratory indicators between the two groups.

Variables^†^	CLS (*n* = 55)	Non-CLS (*n* = 55)	*P*-value
S/T (%)	9.0 (7.6, 11.1)	7.1 (6.3, 8.5)	<0.0001
C-reactive protein (mg/L)	20.0 (8.0, 35.0)	8.0 (8.0, 21.0)	0.040
Serum albumin (g/L)	23.2 (20.5, 25.0)	28.2 (25.6, 31.5)	<0.0001
CLI	8.5 (3.4, 14.3)	3.2 (2.5, 7.1)	<0.0001
Procalcitonin (ng/ml)	7.0 (0.5, 27.6)	4.7 (3.0, 10.8)	0.082
White blood cell count (10^9^/L)	11.7 (7.6, 17.3)	14.7 (7.4, 22.5)	0.411
Serum calcium (mmol/L)	2.0 (1.9, 2.2)	2.0 (1.9, 2.3)	0.593
Red blood cell distribution width (%)	17.8 (16.5, 20.0)	16.9 (16.0, 1.2)	0.098
Coagulation disorders, *n* (%)	25 (45.4)	15 (27.3)	0.047
Blood lactate (mmol/L)	4.3 (2.6, 7.2)	3.1 (2.2, 4.2)	0.014
Blood glucose (mmol/L)	8.0 (6.3, 11.1)	6.1 (5.2, 7.8)	<0.0001

^†^ Variables as number (*n*%) or median [inter-quartile range].

**Figure 1:**
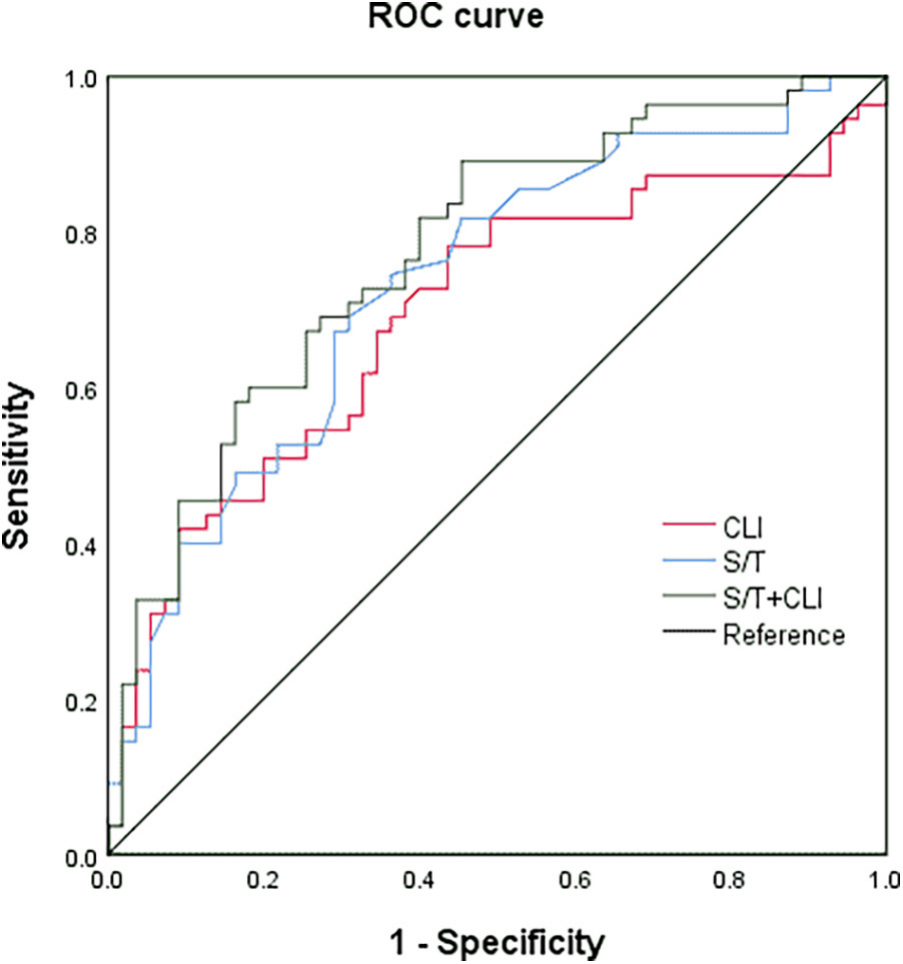
The receiver operating characteristic curve (ROC) of the S/T, CLI, and the combined variables for predicting complicated with CLS in neonatal sepsis.

The significant factors identified above were further analyzed using multivariate logistic regression, with CLS as the dependent variable. The results showed that both S/T and CLI were independently associated with an increased risk of CLS. Specifically, S/T and CLI were positively correlated with CLS, with OR of 1.309 (95% CI: 1.068–1.605, *P* = 0.010) and 1.060 (95% CI: 1.001–1.124, *P* = 0.047), respectively (Table 3). Furthermore, ROC analysis (Figure 1 and Table 4) was conducted to assess the diagnostic accuracy of S/T and CLI for predicting CLS. The AUC for S/T was 0.734, with an optimal cut-off value of 8.1%, sensitivity of 0.673, and specificity of 0.709. The AUC for CLI was slightly lower at 0.693, with an optimal cut-off of 3.3, sensitivity of 0.782, and specificity of 0.564. When both S/T and CLI were combined, the AUC increased to 0.775, with higher sensitivity (0.818) and moderate specificity (0.600). These findings suggest that both S/T and CLI are valuable predictors of CLS, and their combined use improves the predictive accuracy the occurrence of CLS in neonatal sepsis.

**Table 3:** Multivariate logistic regression analysis of the significant influence factors.

Variables	B	SE	Wald *χ*^2^ value	*P*-value	OR value	95% CI for Exp (B)	
						Lower limit	Higher limit
S/T (%)	0.269	0.104	6.722	0.010	1.309	1.068	1.605
CLI	0.059	0.030	3.951	0.047	1.060	1.001	1.124
Coagulation disorders	0.842	0.487	2.990	0.084	2.321	0.894	6.028
Blood lactate	0.061	0.075	0.672	0.412	1.063	0.918	1.231
Blood glucose	0.165	0.093	3.175	0.075	1.180	0.984	1.415

**Table 4:** The value of the S/T, CLI and combined variables for predicting complicated with CLS in neonatal sepsis.

Variables	AUC (95% CI)	Sensitivity	Specificity	Youden index^†^	The optimal cut-off value
S/T (%)	0.734 (0.640–0.827)	0.673	0.709	0.382	8.1
CLI	0.693 (0.593–0.794)	0.782	0.564	0.346	3.3
Combined (S/T + CLI)	0.775 (0.688–0.861)	0.818	0.600	0.418	

^†^ The Youden index = sensitivity + specificity-1.

### 3.4 Prognosis assessment for CLS

The neonatal sepsis complicated by CLS group was divided into two subgroups: the death group and the survival group. There were no significant differences in gestational age, birth weight, sepsis onset age, S/T, or CLI between the two groups (*P* > 0.05). However, the onset time of generalized edema was earlier in the death group [2 (2, 3) vs. 3 (3, 4), *P* = 0.001] after admission. The incidence of negative fluid balance within 7 days of onset was significantly higher in the survival group compared to the death group (71.1% vs. 0%, *P* < 0.01). The duration of mechanical ventilation and hospital stay were significantly longer in the survival group (*P* < 0.05), which may be attributed to the higher urgency, severity, and shorter survival times of the infants in the death group (Table 5).

**Table 5:** Comparison of the survival and death group in CLS group.

Variables	Survival group (*n* = 45)	Death group (*n* = 10)	*P*-value
Gestational age (weeks)	33.0 (30.1, 37.1)	34.6 (30.7, 38.2)	0.585
Birth weight (g)	2,030 (1,350, 2,790)	2,310 (1,510, 3,150)	0.407
Sepsis onset age (days)	1.0 (1.0, 1.0)	1.0 (1.0, 3.5)	0.055
S/T (%)	9.0 (7.8, 11.1)	8.8 (7.0, 11.6)	0.585
CLI	8.5 (3.3, 14.3)	9.3 (4.1, 19.0)	0.348
Generalized edema onset time (post-admission), (days)	3.0 (3.0, 4.0)^a^	2.0 (2.0, 3.0)	0.001
Negative fluid balance^†^ (within 7 days of onset), *n* (%)	32 (71.1)	0 (0.0)	<0.010
Duration of mechanical ventilation (days)	11.0 (6.5, 28.5)	3.5 (2.0, 6.5)	0.001
Duration of hospitalization (days)	28.0 (17.0, 56.5)	3.5 (2.0, 6.5)	<0.0001

^a^ Data missing item, *n* = 37.

^†^ Negative fluid balance: the amount of intake plus the infusion was less than the urine volume.

## 4 Discussion

The morbidity and mortality of CLS in neonates remain underexplored, with limited reports available in the literature. Lin et al. reported an incidence of CLS ranging from 0.3% to 1.62%, with a higher prevalence in males, and a mortality rate between 23.5% and 30.6% (15). In our study, we observed a mortality rate of 18.2% in the CLS group, with 70.9% of cases being male. Neonatal CLS is frequently secondary to sepsis, especially when excessive inflammation accelerates vascular injury and capillary leakage (16). Our study demonstrated that 85.5% of the neonates with CLS exhibited systemic edema. The development of edema in these infants is not merely a consequence of capillary permeability alterations but is also closely associated with altered fluid regulation mechanisms. In our study, elevated lactate levels were significantly associated with CLS (*P* < 0.05). Insufficient oxygen delivery to tissues may further exacerbates capillary permeability and systemic inflammation (17). Moreover, neonates with CLS commonly present with coagulopathy, thrombocytopenia, and pulmonary hemorrhage, likely due to endothelial cell injury and subsequent activation of the coagulation, complement, and fibrinolytic system (18, 19). Multivariate analysis revealed that blood lactate, blood glucose, along with coagulation disorders, were not independent predictors for the occurrence of CLS. This highlights the complexity of CLS pathophysiology and suggests that other, yet unidentified, mechanisms may contribute to its development in septic neonates.

The risk factors, diagnostic markers, and prognosis of CLS have become increasingly important in neonatal care. Despite significant progress, there remains no clinical method to directly monitor capillary leakage (8). Therefore, a comprehensive evaluation incorporating clinical manifestations, imaging techniques, and biomarkers is essential for assessing the occurrence, progression, and prognosis of CLS. The most recent research has proposed that the thoracic fluid content (TFC) plays a key role in the early prediction of secondary CLS in children after cardiopulmonary bypass (CPB), and it measured via electrical bioimpedance to monitoring fluid status in critically ill patients. However, the routine clinical application is limited in most medical institutions due to the lack of specialized equipment (20). Chest radiograph aids in locating the primary septic source and evaluating disease progression. CLS secondary to sepsis is often accompanied by acute respiratory distress syndrome (ARDS), pneumonia, and pulmonary exudative changes, making routine chest radiography necessary. Therefore, S/T, measuring subcutaneous soft tissue thickness via chest radiograph, is objective, readily accessible, as well as does not involve additional radiation exposure. In 2003, Sonntag et al. established a threshold S/T ratio of 12.6% for diagnosing CLS in very low birth weight infants, with edema becoming visible at S/T > 15% (9). While their study focused on diagnostic thresholds, our research aimed to predict the risk of CLS before the onset of overt edema. We found that a S/T ratio greater than 8.1% was positively correlated with neonatal sepsis complicated by CLS, with a sensitivity of 67.3% and a specificity of 70.9%. This suggests that the S/T ratio may serve as a useful early predictor for the risk of CLS in septic neonates, providing clinicians with a potentially valuable tool for early intervention. Additionally, the CLI, which integrates biomarkers such as CRP and albumin, has been proposed as an indicator of disease severity and prognosis. Elevated CRP reflects the extent of inflammation, while decreased albumin levels are indicative of capillary leakage and nutritional status (5, 21–23). A recent study has indicated that the loss of non-albumin plasma proteins (NAPP) is an early and important indicator of CLS persistence and progression to MOF. However, its clinical application is less widespread than albumin, and remains underexplored in CLS, warranting further validation (24). In our study, we found a positive correlation between CLI and neonatal sepsis complicated by CLS. Specifically, a CLI > 3.3 was associated with an increased risk of CLS in septic neonates, with a sensitivity of 78.2% and specificity of 56.4%. When combined with the S/T ratio, the predictive accuracy of CLS improved, as a sensitivity of 81.8%. This indicates that the combined application of S/T ratio and CLI provides a more reliable prediction of CLS in neonates with sepsis, suggesting that both indicators together may improve early risk stratification and guide therapeutic interventions.

Managing CLS in neonates remains a challenge, particularly in the context of sepsis. Our study found that 90.9% of infants with CLS received blood products, which were employed to replenish blood volume, correct anemia, increase platelet counts, and improve coagulation. This finding is consistent with previous research suggesting that blood products and colloids may play a dual role—not only in correcting blood volume deficits but also in facilitating capillary repair and reducing permeability (25). Recent independent studies have indicated the efficacy of 3% NaCl and aminophylline for neonatal CLS (7, 26). However, the clinical application of these interventions must be carefully, the potential effects of which are intriguing and warrant further investigation. During the “leakage phase” of CLS, aggressive fluid resuscitation is necessary to correct hypovolemic shock, as insufficient fluid volume can lead to circulatory collapse (27). However, this approach must be carefully modulated in the subsequent recovery phase. Once fluid begins to return to the capillaries, there is a risk of fluid overload, which can lead to organ edema, particularly in the lungs, and potentially fatal complications. Previous studies have shown that continuous positive fluid balance is correlated with increased mortality rates (28). Similarly, Yang et al. proved that achieving a negative fluid balance within seven days of CLS onset was associated with improved outcomes, including a lower incidence of multiple organ dysfunction, reduced need for mechanical ventilation, and a shorter duration of hospitalization (29). The challenge lies in managing the fluid balance meticulously to prevent the development of acute pulmonary edema and MOF.

Furthermore, the timing of systemic edema onset plays a crucial role in determining the prognosis of neonates with sepsis complicated by CLS. Our data indicate that an earlier onset of systemic edema and a failure to achieve a negative fluid balance within seven days are both associated with poor outcomes. Notably, 71.1% of survivors achieved negative fluid balance within this timeframe, in contrast to none in the non-survivor group. This suggests that early identification of CLS, coupled with a targeted fluid restriction regimen, could improve neonatal outcomes. Such an approach would involve careful fluid management to prevent excessive fluid overload while maintaining stable blood pressure, possibly through permissive hypotension—a strategy that has been advocated in several studies to reduce the risk of fluid accumulation and mitigate the impact of capillary leakage (2, 5). In light of these findings, it becomes evident that optimal fluid management is a cornerstone of successful CLS treatment. Given the delicate nature of fluid balance in critically ill neonates, particularly those with sepsis, a personalized, data-driven approach is essential. Clinicians should consider not only the severity of capillary leakage but also the timing of fluid interventions to minimize adverse outcomes. Future studies should aim to refine fluid management protocols and investigate the role of indicators, such as CLI and S/T ratio, in guiding fluid therapy. Additionally, exploring potential interventions may provide novel approaches for improving the prognosis of neonates with CLS.

## 5 Limitation

This study has several limitations. First, being a single-center, retrospective study with a relatively small sample size, its findings may not be fully generalizable. Future research should focus on multicenter, prospective studies with larger cohorts to validate and expand upon our findings. Second, the retrospective design introduces the possibility of unmeasured confounding factors, despite our efforts to control for bias using propensity score matching. To strengthen the evidence, future studies should adopt prospective cohort designs with comprehensive data collection and long-term follow-up, enabling a clearer understanding of causal relationships and late-onset complications. Third, the physiological characteristics of full-term infants and preterm infants are different, and the following work needs to further analyze respectively. Finally, the identification of additional markers or the combination of multiple predictive factors could enhance the accuracy of risk prediction for CLS in neonatal sepsis.

## 6 Conclusion

Neonatal sepsis complicated by CLS is associated with high mortality, emphasizing the need for early predictive markers. This study shows that combining the S/T ratio with the CLI effectively predicts CLS risk in septic neonates, with higher values indicating an increased risk. While S/T and CLI are useful for risk prediction, they do not assess prognosis. Early onset of systemic edema and failure to achieve a negative fluid balance within seven days were identified as poor prognostic factors. These findings highlight the importance of proactive fluid management to improve outcomes. Further large-scale, prospective studies are needed to validate these results and refine clinical strategies for this vulnerable patient group.

## Data Availability

The original contributions presented in the study are included in the article/Supplementary Material, further inquiries can be directed to the corresponding author.
