# Supplementary material for: The clinical application of subcutaneous thoracic ratio and capillary leakage index on the occurrence of capillary leak syndrome in neonates with sepsis
Source: Front Pediatr. 2025 Jul 7;13:1603378. doi: 10.3389/fped.2025.1603378 (PMC12277306; doi:10.3389/fped.2025.1603378)
Supplement: Supplementary file 1 [file Table1.docx]

***Supplementary Material***

| **Table S1. Comparison of clinical manifestations and comorbidities** | | | |
| --- | --- | --- | --- |
| Variables^*^ | CLS (n=55) | Non-CLS (n=55) | *P*-value |
| Infection sites, n (%) |  |  |  |
| pneumonia | 53 (96.4) | 51 (92.7) | 0.675 |
| gastrointestinal infection | 11 (20.0) | 3 (5.4) | 0.045 |
| intracranial infection | 8 (14.5) | 7 (12.7) | 0.781 |
| Blood culture, n (%) |  |  |  |
| Gram-positive bacteria, n (%) | 6 (11.5) ^b^ | 1 (2.1) ^c^ | 0.145 |
| Gram-negative bacteria, n (%) | 3 (5.8) ^b^ | 2 (4.2) ^c^ | 0.713 |
| Hemorrhage, n (%) |  |  |  |
| pulmonary hemorrhage | 29 (52.7) | 7 (12.7) | <0.0001 |
| gastrointestinal bleeding | 6 (10.9) | 12 (21.8) | 0.122 |
| intracranial hemorrhage | 23 (41.8) | 18 (32.7) | 0.324 |
| Thrombopenia, n (%) | 9 (16.4) | 2 (3.6) | 0.026 |
| Acute respiratory distress syndrome, n (%) | 36 (65.4) | 29 (52.7) | 0.175 |
| Hyperbilirubinemia, n (%) | 39 (70.9) | 37 (67.3) | 0.680 |
| Hypokalemia, n (%) | 9 (16.4) | 0 (0.0) | 0.003 |
| Hyponatremia, n (%) | 4 (7.3) | 0 (0.0) | 0.118 |
| Renal insufficiency, n (%) | 14 (25.4) | 12 (21.8) | 0.654 |
| Liver function damage, n (%) | 10 (18.2) | 4 (7.3) | 0.153 |
| Myocardial damage, n (%) | 20 (36.4) | 12 (21.8) | 0.093 |
| Shock, n (%) | 24 (43.6) | 6 (10.9) | <0.0001 |
| Critical illness^†^, n (%) | 41 (74.5) | 20 (36.4) | <0.0001 |
| ^*^Variables as number (n%), b data missing item, n=52, c data missing item, n=48.  ^†^The inclusion criteria for critically illness were defined according to the Neonatal Critical Illness Score (NCIS). In: Shao XM, Ye HM, Qiu XS, editors. *Practice of Neonatology.* 5th edition. Beijing: People's Medical Publishing House; 2019. p. 292-294. | | | |

| **Table S2. Comparison of the treatment and outcome between the two groups** | | | |
| --- | --- | --- | --- |
| Variables^*^ | CLS (n=55) | Non-CLS (n=55) | *P*-value |
| Mechanical ventilation, n (%) | 54 (98.2) | 42 (76.4) | 0.002 |
| Duration of mechanical ventilation (days) | 8.5 (5.0, 22.5) | 5.0 (2.0, 14.2) | 0.006 |
| Administration of blood products, n (%) | 50 (90.9) | 30 (54.5) | <0.0001 |
| Administration of albumin (g) | 11.6 (5.4, 18.0) | 2.4 (0.0, 7.6) | <0.0001 |
| Administration of vasoactive drugs, n (%) | 47 (85.4) | 22 (40.0) | <0.0001 |
| Duration of hospitalization (days) | 24.0 (13.7, 54.5) | 27.0 (12.7, 48.7) | 0.848 |
| Mortality, n (%) | 10 (18.2) | 4 (7.3) | 0.153 |
| ^*^Variables as number (n%) or median [inter-quartile range]. | | | |


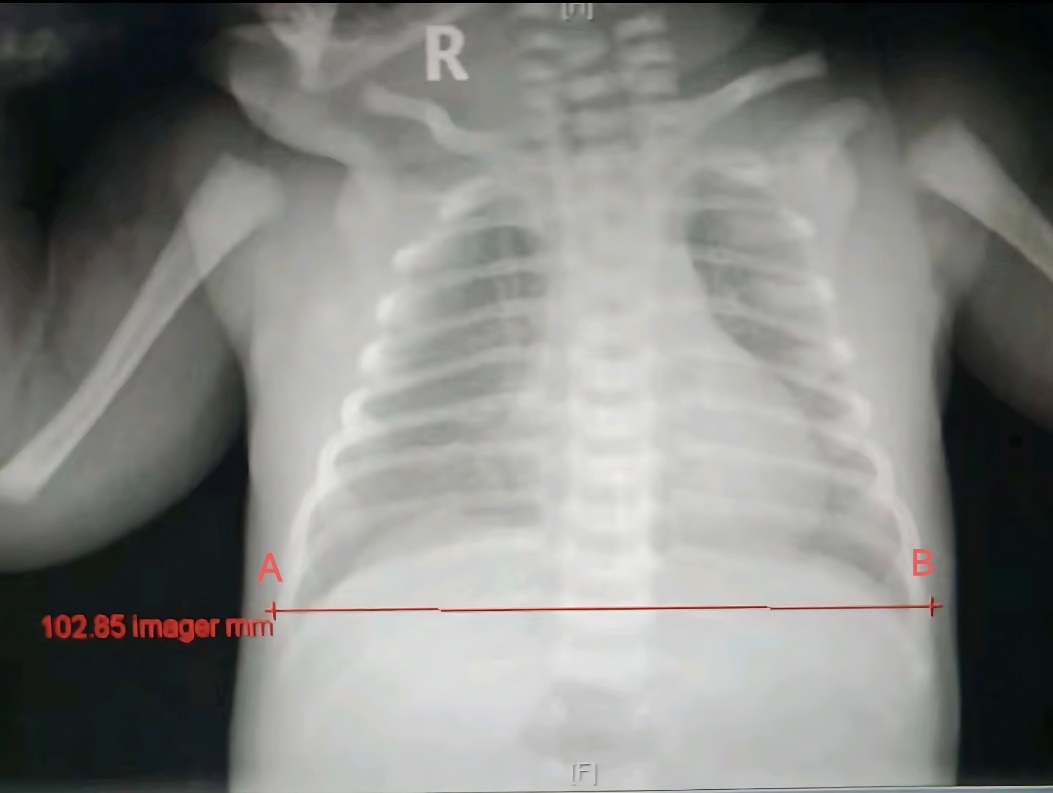


**Figure S1**. The distance of the outer edge of the 8th rib





**Figure S2.** Total thoracic diameter at the same position
